# TP or Not TP? Successful Comparison of Two Independent Methods Validates Total Phosphorus Inference for Long-Term Eutrophication Studies

**DOI:** 10.1021/acs.est.4c01816

**Published:** 2024-04-19

**Authors:** Madeleine Moyle, John Boyle, Helen Bennion, Richard Chiverrell

**Affiliations:** † Department of Geography and Planning, University of Liverpool, 74 Bedford St South, Liverpool L69 7ZT, United Kingdom; ‡ Department of Geography, University College London, North-West Wing, Gower Street, London WC1E 6BT, United Kingdom

**Keywords:** phosphorus, total phosphorus reconstruction, lake sediment, geochemistry, diatom, eutrophication

## Abstract

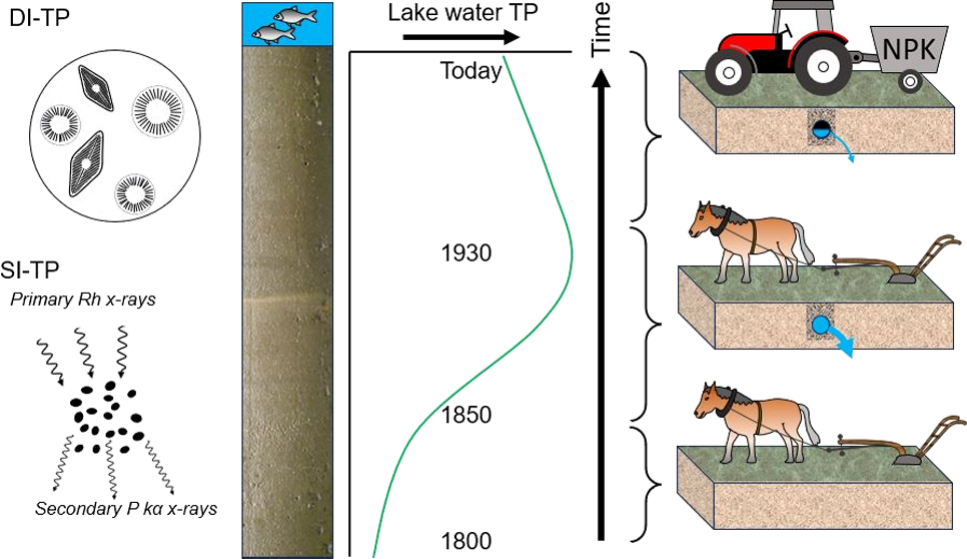

Validating paleo total phosphorus (TP) inference methods over long time scales is essential for understanding historic changes in lake P supply and the processes leading up to the present-day global lake eutrophication crisis. Monitored lake water TP time series have enabled us to identify the drivers of eutrophication over recent decades. However, over longer time scales, the lack of reliable TP inference means our understanding of drivers is speculative. Validation of lake water TP reconstruction, therefore, remains the “ultimate aim” of eutrophication studies. Here, we present the first critical comparison of two fully independent paleo TP inference approaches: the well-established diatom method (DI-TP) and a recently developed sediment geochemical method (SI-TP). Using lake sediment records from a small eutrophic U.K. lake (Crose Mere), we find a statistically significant agreement between the two inferred TP records with greater than 60% shared variance. Both records show identical timings, with a 19th century acceleration in TP concentration and subsequent declines following a peak in 1930. This significant agreement establishes the validity of long-term paleo TP inference for the first time. With this, we can now test assumptions and paradigms that underpin understanding of catchment P sources and pathways over longer time scales.

## Abstract 2

Monitoring records provide the data needed to understand what is driving the global lake eutrophication crisis over recent years; however, over longer time scales, we rely on contested inference methods. This study provides the first independent validation of long-term lake water total phosphorus inference.

## Introduction

Over the last century, human intensification of the terrestrial phosphorus (P) cycle has caused more than 1.4 × 10^12^ kg of P to be lost to the global aquatic environment.^1^ These excessive P loads have substantially impacted global water resource value, aquatic biodiversity, and ecosystem stability.^2−4^ Despite concerted efforts to mitigate these impacts, 44% of European lakes are failing to meet water quality targets,^5^ and the US economy loses over $2.2 billion a year as a result of human-induced eutrophication.^6^ Furthermore, interactions between climate breakdown and human pressures point to sustained decreases in the resilience of freshwater ecosystems and the likely intensification of ecosystem damage and economic loss.^7−10^

This present-day global eutrophication crisis follows a long history of human activities that have increased P supply to lakes^11,12^ and a trend toward eutrophic conditions from ca. 1850 onward.^13−16^ Some of the most substantial impacts on lake water total phosphorus (TP) concentrations are seen in lowland landscapes that are either urban or high-intensity agricultural catchments.^17^ In lowland rural catchments, mid-20th century agricultural intensification and the widespread adoption of chemical fertilizers are widely regarded as major causes of lake eutrophication,^3,18−20^ particularly through cumulative “legacy P” effects.^21^ However, Withers et al.,^3^ Naden et al.,^22^ and Bell et al.^23^ also show that wastewater remains a substantial source of P to lakes, with an estimated 14.8 × 10^6^ kg P supplied by domestic wastewater to U.K. lakes in 2010.^22^ Uncertainty persists over the relative importance of the major P sources in these landscapes, particularly over the last few centuries. It is therefore critical that we fully understand the long-term (here referring to centennial scales) interaction between P cycling, human activity, and natural aquatic systems to ensure successful and cost-effective management interventions in the face of future climate uncertainty,^24^ particularly as increased global temperatures could confound recovery of lakes from eutrophication.^25^ For this, the reliable identification, quantification, and apportionment of P sources in anthropogenic landscapes are essential for reversing the trend of freshwater eutrophication.^3,19^

Assumptions regarding dominant P sources over the last few centuries cannot be tested using lake water monitoring data because the earliest time series do not extend far enough back into the past. For example, the earliest continuous TP records in the U.K. date from the late 1960s and early 1970s, such as Loch Leven in Scotland,^26^ and Esthwaite Water and Windermere South Basin in England;^27^ however, such time series are rare. Landscape P models, such as INCA-P^28^ and PSYCHIC,^29^ although valuable to our understanding of P source apportionment, cannot fill this data gap because of the lack of suitable data with which to validate model outputs over centennial time scales. Without long-term monitoring data, it is not possible to test long-term model predictions^30^ or evaluate model performance across more substantial system changes. Current and future monitoring schemes will go some way to resolve this but cannot address the gap in empirical historical data and, therefore, cannot truly resolve the uncertainty surrounding the historical drivers of lake eutrophication.

A solution is offered by paleolimnology, which has the potential to provide the long-term data needed to test empirically which anthropogenic sources have controlled P fluxes and lake water TP concentrations, using the transfer function approach.^31−35^ Diatom inferred TP (DI-TP) has been shown to successfully reproduce spatial patterns present in modern ecological training sets,^31−33^ to reproduce short, monitored records,^36−38^ and to infer temporal records consistent with historical application of export coefficient models.^39^ More recently, an alternative inference method has been developed that uses sediment geochemical records. The sediment-inferred TP (SI-TP) model^40^ is based on fundamental limnological principles about steady-state lake P mass balances^41^ and uses a lake P retention coefficient (*R*_P_) and apparent P burial rates from dated sediment cores. Like DI-TP, this method has shown good agreement with monitored lake water TP data.^40,42^

However, over longer time scales, neither DI-TP nor SI-TP has been validated owing to a lack of sufficiently long lake water TP time series, and consequently, all long-term paleolimnological TP inference records using these methods remain unproven. Additionally, the use of DI-TP has been criticized on theoretical grounds,^43^ and specifically Juggins et al.^44^ have questioned the application of DI-TP to pre-1950 records owing both to a hypothesized change in the diatom–phosphorus relationship and the absence of independent validation. As neatly summarized by Davidson and Jeppesen^45^ in a review of paleolimnological approaches to assessing the impact of eutrophication on lake systems: “what remains moot is the veracity and utility of precise quantitative inference of past nutrient levels [from DI-TP].” They find that paleo TP inference is increasingly used and cited by researchers beyond the paleolimnology community, making validation imperative. Validating paleo TP inference methods over the longer term is essential for understanding historic changes in lake P supply and the impacts leading up to the present-day eutrophication crisis. Monitoring data cannot resolve this, but multiple fully independent inference methods can. Diatom communities and sedimentary P are controlled by independent factors. Sedimentary P is dependent on autochthonous and allochthonous processes, forming both organic and inorganic fractions that can be variably retained in the sedimentary profile.^46^ In contrast, diatom presence and diversity are biological responses to the physiochemical properties of their habitat, moderated by stochastic ecological interactions.^47^ Showing that DI-TP and SI-TP can reconstruct the same TP history, therefore, constitutes a mutual validation because an agreement between the two methods must point to a common controlling factor, which can only be the lake water P concentration. Such verification of paleo TP inference will ultimately enable the debate over drivers of long-term eutrophication to be resolved.

Our study presents the first critical comparison of the DI-TP and SI-TP methods. Here, we aim to (1) compare the DI-TP and SI-TP records from a single site to test for an agreement and, if an agreement is found, (2) examine the timing and intensity of change in the TP record to identify the controls over historic lake P supply.

## Material and methods

### Study site

**Figure 1:**
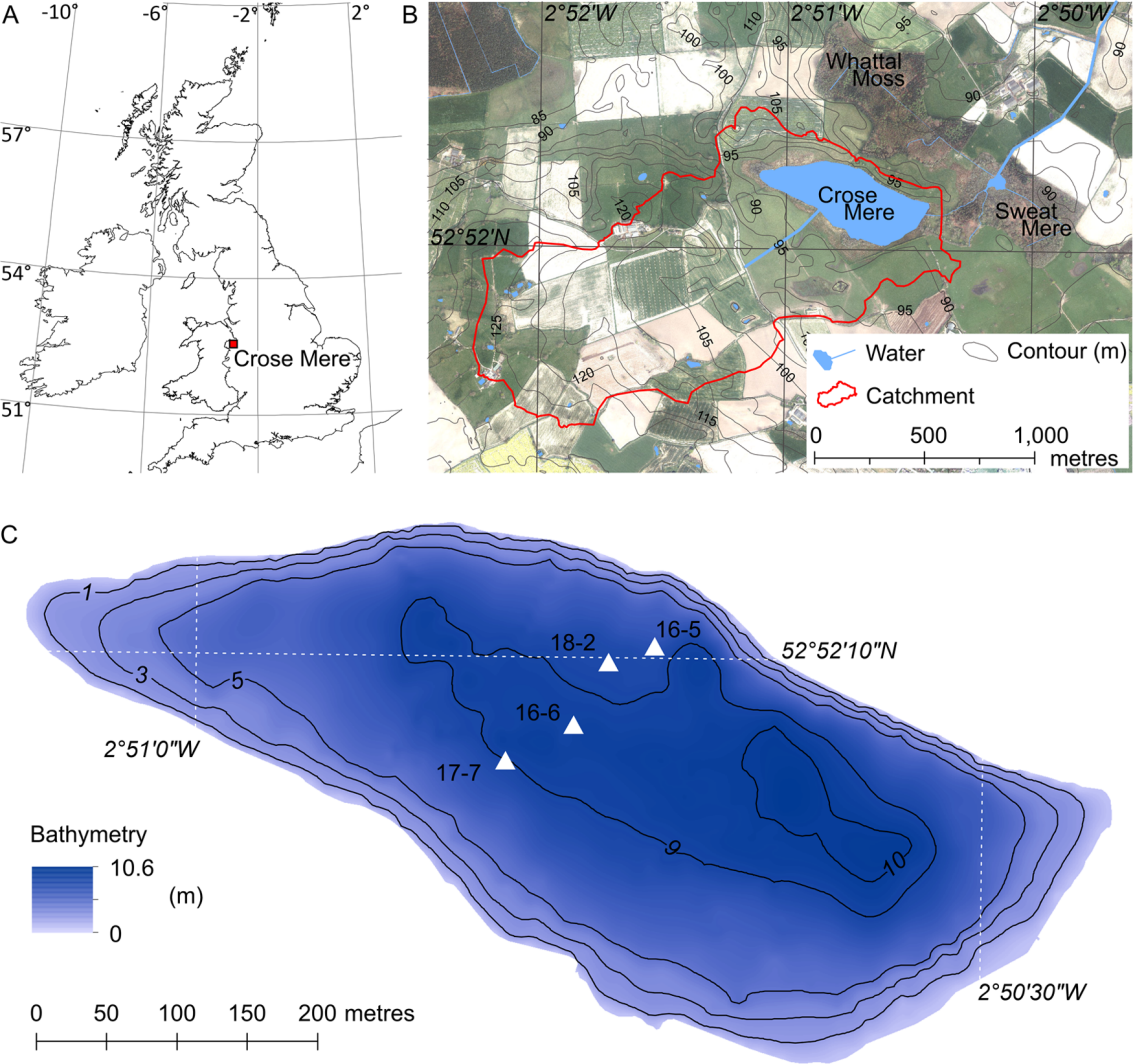
“The location of the study site. (A) Location of Crose Mere, U.K., (B) character of the catchment, and (C) lake bathymetry and sediment cores used in this study. Digimap licensed data Crown copyright and database rights 2023 Ordnance Survey (100025252) and Aerial Digimap Getmapping Plc.”

In this study, we focus on Crose Mere, a small (0.154 km^2^), relatively shallow (max. depth = 9.3 m, mean depth = 6.3 m) eutrophic lake located in a predominantly agricultural lowland (88 m above datum) catchment (1.72 km^2^) in Shropshire, U.K. (Figure 1). The lake has an outflow stream and one intermittent surface inflow stream. The site is one of the Shropshire-Cheshire meres, a group of over 60 lowland lakes of high ecological importance that have long been identified as some of the most nutrient-rich water bodies in the U.K.^48−50^ Crose Mere was selected as its catchment is an exemplar of the typical land use of lowland agricultural sites across Europe.

### Sampling Strategy and Sediment Composition

For the sedimentary geochemical P records, four profiles were taken between 2016 and 2018 (Figure 1). Each profile consists of a 75 mm × 1.5 m core taken with a hand-percussive Russian core deployed from an anchored floating platform, with the sediment–water interface captured using an 80 mm diameter gravity core.^51^ The Russian cores were sliced at 1 cm intervals (2 cm in the case of CRO18). Gravity cores were extruded and sliced at 0.5 cm intervals (1 cm intervals in the case of CRO18-GC). All samples were freeze-dried, and bulk sediment geochemistry was measured using a Spectro Xepos 3 ed-XRF analyzer and corrected for organic content using the accompanying software, with organic content measured using near-infrared diffuse reflectance spectroscopy (NIRS) following the method of Russell et al.^52^ The sedimentary diatom record is from a single ^210^Pb-dated core taken in 1993 from the deepest point of the lake. Full details of this sediment core can be found in Bennion et al.^49^

### Chronology

For the 2016–2018 cores, ages for the uppermost sediment layers were obtained by ^210^Pb dating carried out by P.G. Appleby and G.T. Piliposian at the Environmental Radioactivity Research Centre, University of Liverpool. ^210^Pb dating was carried out on dried sediment samples from three gravity cores. Subsamples from each core were analyzed for ^210^Pb, ^226^Ra, ^137^Cs, and ^241^Am by direct γ assay using Ortec HPGe GWL series well-type coaxial low background intrinsic germanium detectors.^53^ To secure the chronology of the earlier part of the records presented here, five plant macrofossil samples were submitted for radiocarbon analysis. Four macrofossil samples were prepared for graphite at the NERC Radiocarbon Facility-East Kilbride and passed to the Scottish Universities Environmental Research Centre Accelerator Mass Spectrometry (SUERC AMS) Laboratory for ^14^C analysis. The fifth sample was prepared for graphite at the NERC Radiocarbon Facility-East Kilbride and passed to the Keck Carbon Cycle AMS Facility, University of California, Irvine, and analyzed by AMS at low current, requiring the expertise of Dr Xiaomei Xu. A master chronology was produced using Bacon (v2.5.0)^54^ in R (v4.0.3) and transferred to all cores using geochemical horizon matching. Priors were set as accumulation rate (γ distribution) mean = 30 yr cm^–1^ and shape = 1.5 and memory (β distribution) mean = 0.2 and shape = 15. Radiocarbon dates were calibrated using IntCal20.^55^

### Calculation of SI-TP

The SI-TP records were calculated from the sediment geochemical records following the method of Moyle and Boyle,^40^ where
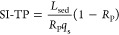
1The P load to the sediment (*L*_sed_) was calculated for each core using the sedimentary P concentration, sediment mass accumulation rate, and the model of Håkanson^56^ to correct for sediment focusing. Areal water loading (*q*_s_) was calculated using modeled inflow values (for the period 2003–2018) adjusted to monitored inflow values measured by the authors between 2016 and 2018. For this, lake outflow for ungauged years was estimated by regressing monitored discharge data (2016–2018) onto a gauged river flow record from the same catchment (Roden at Rodington, National River Flow Archive site 54,016, https://nrfa.ceh.ac.uk/data/station/info/54016), giving a value of 2.79 m yr^–1^. The P retention coefficient (*R*_P_) was calculated using *L*_sed_ and outflow P loading (*L*_out_), both corresponding to the 2003–2018 monitoring period. *L*_out_ was calculated using the 2003–2018 modeled flow rates and monitored TP values for the same period.^57^ This approach to calculate *R*_P_ effectively anchors the top of the SI-TP record to the average 2003–2018 monitored TP value. Further details and discussion of the calculations used can be found in Moyle and Boyle,^40^ and full details of the DI-TP record can be found in Bennion et al.^49^

To correct for the stationary P peak present in the top of the sediment geochemical records, the diagenesis model of Penn et al.^58^ was applied to the SI-TP records, following the method developed by Boyle et al.^42^ The Penn model^58^ accounts for the rate and extent of P loss from freshly deposited sediment by distinguishing a stable P fraction from an unstable P fraction that undergoes first-order decay. Such stationary peaks are temporary phenomena, diffusing to the water column at decadal time scales,^42^ and are diagenetic effects that are not part of the depositional P signal interpretable via SI-TP. Correcting for this diagenetic effect (here, referred to as Penn-corrected SI-TP), therefore, allows the affected portion of the record to be analyzed and compared with the DI-TP profile.

To enable comparison between the SI-TP and DI-TP profiles and overcome issues with irregular spacing in paleolimnological time series data,^59^ a generalized additive model (GAM) was fitted to the records using the mcgv package (v1.8–41)^60^ in R (v4.0.3).

## Results and Discussion

**Figure 2:**
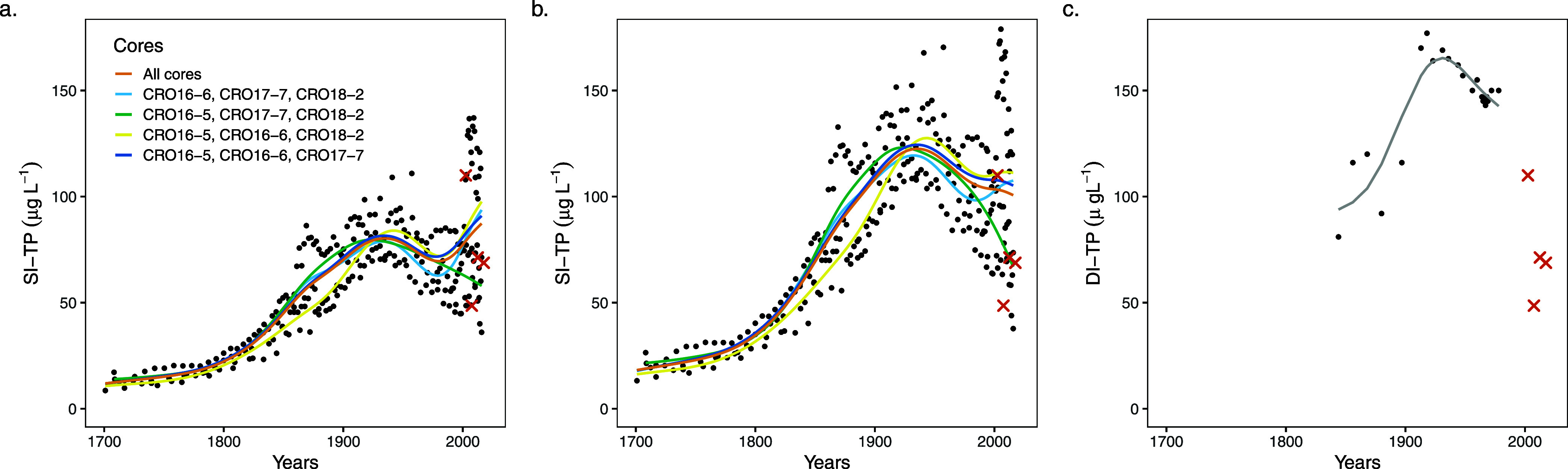
“Inferred TP records compared with measured TP data (5-year means; orange crosses). Paleo inferred (black dots) lake water TP values with fitted GAMS (lines) for (a) uncorrected SI-TP values, (b) SI-TP values corrected for diagenesis (Penn-corrected SI-TP), and (c) DI-TP.”

Figure 2 shows the reconstructed lake water TP records based on both inference methods and measured lake water TP for the site. The first curve presents the unmodified SI-TP model output (Figure 2a), and a second (Figure 2b) shows the Penn-corrected SI-TP. The DI-TP record (Figure 2c) is truncated in the mid-19th century due to poor diatom preservation in the lower part of the core.^49^

All three records (Figure 2) commence with a rising trend peaking at ca. 1930 (GAM peaks: 1931 for DI-TP, 1928 for mean SI-TP, and 1934 for Penn-corrected mean SI-TP). This pattern is not surprising, given the land use history of the site because rapidly increasing recent TP concentrations are characteristic of lowland agricultural landscapes^11^ and increasingly eutrophic conditions are widespread across European lakes from ca. 1850.^13−15^ From the peak at 1930 and until ca. 1980, the DI-TP and Penn-corrected SI-TP records both show clear falling trends (Figure 2b,c), with the uncorrected SI-TP record showing a falling trend interrupted by the stationary peak and, therefore, becoming uninterpretable. After ca. 2000, both SI-TP records show a bimodal population of modeled values, a phenomenon not apparent earlier in the record, and attributable to heterogeneity in the stationary P peak across the four cores.^40,42^ Despite the partial recovery in water quality during the second half of the 20th century following the 1930s peak, inferred TP concentrations remain higher than preindustrial levels—a phenomenon widely reported in lake DI-TP records elsewhere.^13^ DI-TP and SI-TP both show sharply rising TP values through the later 19th century, but it is the longer SI-TP record that reveals an acceleration beginning at ca. 1800. It also evidences an initial rise substantially prior to 1850, reinforcing the argument of Bradshaw et al.^61^ and Moyle et al.^11^ that long temporal records are essential for fully understanding eutrophication history.

**Figure 3:**
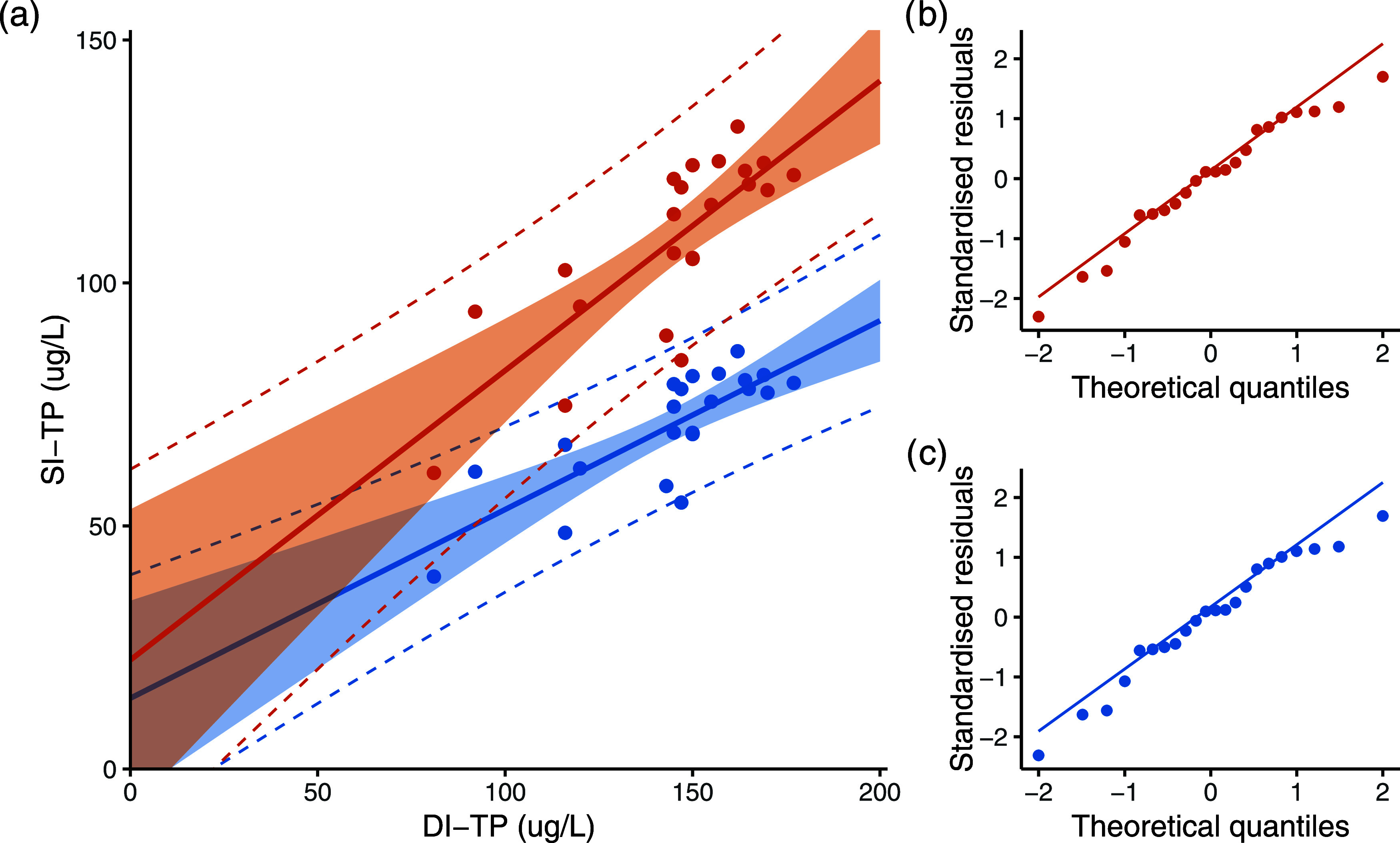
“Correlation of DI-TP values with SI-TP mean values centered on the DI-TP dates. (a) DI-TP against raw SI-TP (blue, *y* = 0.5957*x* + 22.411, *r*^2^ = 0.631, *p* < 0.0005) and Penn-corrected SI-TP (orange, *y* = 0.3884*x* + 14.552, *r*^2^ = 0.6345, *p* < 0.0005) shown with 95% confidence intervals (shaded ribbon) and 95% prediction intervals (dashed lines); (b) normal Q-Q plot for Penn-corrected SI-TP; and (c) normal Q-Q plot for raw SI-TP. Residual distributions are not significantly different from normal and show no association with the fitted value.”

Comparison of the inferred concentrations (Figure 3) finds a statistically significant correlation between DI-TP and SI-TP and greater than 60% shared variance. We expect that this underestimates the true agreement of these two TP approaches owing to the differing nature of signal smoothing inherent in DI-TP and SI-TP models. Figure 3 also shows that regression intercepts are not different from zero, consistent with a similar sensitivity of the two approaches over the full range of TP values.

These two proxies are fundamentally independent, with the diatoms reflecting lake productivity and ecosystem structure and the sediment geochemical P record reflecting the sediment–water P dynamics. Therefore, the strong agreement observed between the DI-TP and SI-TP records points to the existence of a single underlying controlling factor, with the statistically significant association allowing us to reject the possibility of a chance agreement. Given the nature of these two methods, the common controlling factor is most likely to be the lake water P concentration.

It is important to consider here whether this controlling factor is the sum of all P fractions in the lake water, i.e., TP, or some specific P fraction such as soluble reactive P (SRP). For SI-TP, the model is based on the total mass balance of P in the lake–catchment system and, therefore, encompasses all P fractions. This means the model could produce values higher than those measured in lake water TP because it may include a substantial proportion of fast-settling particulate-bound P of terrigenous origin that is not necessarily captured in a monitored lake water TP sample. In contrast, diatoms are responding to biologically available P forms, which may be better represented by SRP.^39,62^ For DI-TP inference, it is assumed that this P fraction is proportional to TP. However, it has been argued that this relationship may have changed during the mid-20th century (1930–1950) due to widespread adoption of chemical fertilizers.^44^ This would be reflected as a change in the relationship between SI-TP and DI-TP in the more recent part of the record. From Figures 2 and 3, we can see that whichever P fraction is driving both SI-TP and DI-TP, it does not change substantially in proportion to TP over the period reconstructed, and we conclude that both methods represent TP. Our successful comparison of SI-TP and DI-TP at Crose Mere, therefore, provides independent validation, a key criterion of Juggins et al.^44^ for reliable application of TP inference. We show that any potential SRP/TP change at the site is not a barrier to the successful implementation of DI-TP reconstruction prior to 1950, and it appears that both inference methods are reliably reconstructing TP at Crose Mere.

SI-TP and DI-TP show almost identical trends; however, the similarity between the records does not extend to their magnitudes (Figure 2), suggesting a bias in one or both of the inferred records. The DI-TP average values are approximately double the uncorrected SI-TP values; the interval weighted mean (mean of decadal means, to correct for variable sampling density) is 136 μg L^–1^ for DI-TP and 68 μg L^–1^ for uncorrected SI-TP. The Penn-corrected SI-TP record has a larger mean of 104 μg L^–1^, bringing it closer to the DI-TP value, and while both records infer values in the hypereutrophic range (>100 μg L^–1^), the corrected SI-TP mean remains substantially lower than the DI-TP mean. For the most recent samples, both DI-TP and SI-TP lie within the range of monitored TP values; however, note that the DI-TP and monitored records do not overlap as the core predates the monitoring data (Figure 2). In the case of the SI-TP data, the interval 2003–2018 is constrained to have identical mean values to monitored TP because of the method used to calculate *R*_P_;^40^ therefore, agreement is expected for the most recent values. However, the corrected SI-TP (Figure 2c) captures the falling trend observed in the monitored data extending beyond the anchor point, which is not caused by the anchoring process. Without a longer monitoring record, it is not possible to test which of the records most accurately reflects the original historical lake water TP values. However, it is possible to reflect on reasons that might lead to systematic bias for either proxy.

Numerical biases in SI-TP could arise from several causes. When driven by an independently estimated *R*_P_, the accuracy of SI-TP depends on correctly inferring mean lake-wide burial rates from a limited number of sediment cores. Here, we have avoided this problem by using a locally calibrated *R*_P_ based on the recent sediment record and the measured lake water TP (see Materials and Methods section), which compensates for any bias in the estimated lake-wide P burial rate.^40^ However, the extent of diagenetic decay of the sedimentary P signal will alter the magnitude of earlier SI-TP values relative to recent values.^42^ Without correcting the inferred values using the Penn et al.^58^ model, the SI-TP model will underestimate TP (Figure 2a,b). For Penn-corrected SI-TP, the assumed magnitude of the stable P fraction will impact the magnitude of the inferred TP values, and an incorrect value used in the Penn model would bias the results.^42^ Bias may also arise because of the use of total sediment P concentrations to capture the whole mass balance, a fundamental principle of the SI-TP model. Inclusion of the terrigenous P fraction may lead to an overestimation of lake water TP, but at Crose Mere, this effect will be minor due to the small inflow stream and, consequently, low terrigenous load.

The nature of the bias in DI-TP records is quite different. Despite stratigraphic trends providing ecologically and environmentally plausible results,^44^ bias in DI-TP relative to corresponding mean measured lake water TP values has long been recognized, with substantial unpredictable site-to-site variation in magnitude. Biases in DI-TP can arise due to site-specific characteristics. Lake water TP has typically been found to explain 8–12% of the variance in the diatom species data.^31−33^ Of the remaining variance, lake maximum depth and measures of base richness (pH, Ca, conductivity) have substantial independent effects,^31,33^ while most variance is unexplained, a fact attributed to the multivariate nature of factors controlling diatom abundance.^31−33^ During the development of calibrations from training sets, it has been necessary to disregard some samples without clear a priori justification, showing the presence of very substantial bias at some lakes.^31−33^ An additional well-recognized potential cause of bias in DI-TP reconstructions relates to the abundance of benthic relative to planktonic taxa, the former tending to have a less direct relationship with lake water TP owing to other influences such as light, substrate type, and grazing pressure.^14,39,63^ This can result in poorer agreement with measured TP when samples are dominated by benthic forms.^44^ However, this is largely a feature of shallow lakes, typically less than 3 m maximum depth, and is not an issue in the case of the Crose Mere diatom record, which, owing to its greater water column depth, is dominated by planktonic taxa such as *Cyclotella spp*., *Stephanodis*c*us spp*., *Aulacoseira spp*., *Fragilaria crotonensis*, and *Asterionella formosa*.^49^ Diatom preservation deteriorated downcore but did not prevent counting above 60 cm (post-1800); hence, while preferential preservation of some taxa over others can affect diatom assemblage data, it is not thought to have strongly influenced the data presented here.

Given the potential for bias in both inference methods, the significant agreement between the records produced by these two fully independent models is remarkable. That both methods have produced records with comparable magnitudes and that the inferred trends are almost identical leads us to conclude that both methods have successfully reproduced historical long-term lake water TP. This is the first time a paleoecological inference method and a sediment geochemical inference method have been critically compared. Our study at Crose Mere is the first successful validation of paleo TP inference and represents a significant milestone in paleo inference. Here, we achieve the “ultimate aim for eutrophication-based studies of lakes [which is] an independent reconstruction (or inference) of phosphorus.”^45^ As Crose Mere is typical of lowland agricultural lakes, and both inferred TP records follow the well-recognized trajectory of eutrophication and partial recovery,^11,13,14,16^ we have no reason to doubt this finding cannot be replicated elsewhere.

With our validated record, we can interrogate the hypothesized drivers of change in long-term lake water TP concentrations. For lowland agricultural lakes, mid-20th century agricultural intensification and the widespread adoption of chemical fertilizers^64^ are widely regarded as the main drivers of eutrophic conditions through “legacy P” effects.^3,18−21^ At Crose Mere, however, we find that the inferred records show rapidly increasing TP concentrations from ca. 1850 (Figure 2), a century in advance of these agricultural changes. In fact, the post 1930 period of agricultural intensification coincides with a gradual decrease in TP concentrations and, therefore, cannot explain the eutrophication trend, leaving no role here for “legacy P.” Instead, a plausible explanation for the post-1850 acceleration in TP at Crose Mere is greater connectivity between the lake and the catchment caused by the installation of field underdrainage and the increase in the number of people connected to water sanitation systems. The 19th century push for land improvement encouraged farmers across the U.K., and more broadly across Europe and North America, to drain their fields to maximize the area of land under production and increase crop quality.^65,66^ In the 1840s, the installation of field underdrainage was accelerated by the mass production of the clay drainage pipe and the increasing availability of government loans.^67^ The peat and marshland in the area around Crose Mere were extensively drained during this period,^68^ and between the 1840s and 1930s, over 75% of the land in Shropshire, the county in which Crose Mere is located, had field drains installed.^69^ The other important change to happen during this period was the early 20th century adoption of flush toilets in rural Britain.^22^ Like the installation of field underdrainage, these new sanitation systems provided a direct connection between catchment P sources and lakes, increasing the supply of P to freshwaters. Our findings at Crose Mere give direct evidence of a TP increase coincident with the 19th century push for land improvement to which early ecological changes have been previously attributed.^16,70^ Based on the timing of changes in TP, the prevailing narrative that “legacy P” effects from the mid-20th century shift to chemical agriculture are the primary drivers of eutrophication at lowland agricultural sites is invalid at Crose Mere. Here, instead, we find connectivity between the lake and the catchment to be the key factor in controlling the historic P supply. To test this finding more widely, particularly at other sites that show early TP increases,^13^ we suggest further studies into the relative importance of P sources over the critical post-1800 time frame are needed.

Our study is the first to establish the validity of long-term paleo TP inference through the comparison of two fully independent methods: DI-TP and SI-TP. Here, we have met the key criterion of Juggins et al.^44^ for reliable application of these methods and achieved the “ultimate aim” of independent TP reconstruction.^45^ We have shown that the acceleration of TP concentrations at Crose Mere occurred from ca. 1850, substantially prior to lake water TP monitoring. Given that many lakes experienced a similar premonitoring increase in TP concentrations,^13,14,16^ paleo inference approaches are the only reliable way to resolve what factors have driven long-term lake eutrophication. With our validation of paleo TP inference approaches, we can now test assumptions and paradigms that underpin our understanding of catchment P sources and pathways over longer time scales. This evidence from the paleo record is critical supporting information for nurturing resilient aquatic systems in the face of future climate uncertainty and other emerging pressures.
